# Constitutional Nephrin Deficiency in Conditionally Immortalized Human Podocytes Induced Epithelial-Mesenchymal Transition, Supported by **β**-Catenin/NF-kappa B Activation: A Consequence of Cell Junction Impairment?

**DOI:** 10.1155/2013/457490

**Published:** 2013-11-24

**Authors:** Gian Marco Ghiggeri, Maddalena Gigante, Armando Di Donato

**Affiliations:** ^1^Department of Nephrology, Gaslini Children Hospital, Genoa, Italy; ^2^Department of Biomedical Sciences, Interdepartmental Research Center BIOAGROMED, University of Foggia, Italy; ^3^Laboratorio di Nefrologia, Istituto G. Gaslini, Largo G. Gaslini 5, 16147 Genova, Italy

## Abstract

The kidney glomerular podocytes are the cellular target of many chronic nephropathies both determined and acquired genetically. Mutations that affected the expression and/or the function of nephrin, a key component of the slit-diaphragm, are often causes of these pathologies. Recent findings showed that murine podocytes could undergo epithelial-mesenchymal transformation (EMT), suggesting new hypotheses about the pathogenesis of glomerular fibrosis. Here, we show that also human podocytes can undergo EMT, but more importantly nephrin ablation itself can trigger this phenotypic transformation. In fact, a model of human podocyte with engineered nephrin deficiency constitutionally expressed high levels of *α*-SMA, vimentin, fibronectin, and other hallmarks of EMT. Since it is known that cell contact abrogation is one of the triggers of EMT, we reasoned that nephrin loss could account for such cell junction disruption and cause the EMT. Therefore, we demonstrated that also normal podocytes could spontaneously undergo EMT if grown in Ca^2+^-free medium, which is known to impair cell contacts. The analysis of the main intracellular signal transduction pathways evidenced some major anomalies consequent with the nephrin abrogation. The most intriguing was the activation of *β*-catenin pathway, which plays a critical role in podocyte ontogenesis as well as in the nephrin expression and EMT regulation. Also other important signaling proteins, like NF-*κ*B, p53, and retinoblastoma protein (RB), showed important activity modifications. Interestingly, most of the above indicated signaling pathway alterations were again reproducible by cell junction rupture, induced by Ca^2+^ deprivation. Finally, immunofluorescence analysis on kidney sections of patients with NS of Finnish type confirmed the constitutive expression of *α*-SMA.

## 1. Introduction

Epithelial-mesenchymal transition (EMT) and its reverse process (MET) are physiological processes operative since the first days of the embryological development [1, 2]. In adults, this phenotypic transition is often just a physiological response to a call for tissue regeneration, as in the case of normal wounds [3, 4]. In fact, the cells deriving from EMT, the myofibroblasts, smooth muscle cells alike, are specialized fibroblasts highly active in the production of extracellular matrix components (collagens, elastin, fibronectin, metalloproteinase, etc.) [5]. Unfortunately, EMT is often exacerbated during pathological events such as organ fibrosis, where the process occurs out of control and is irreversible. Recently, it has been pointed out that this differentiating process could be at the basis of the spreading of many epithelial cancers, since the cells resulting from EMT acquire a much higher mobility potential [6, 7]. Among the many organ pathologies characterized by a fibrotic outcome, the renal nephrotic syndrome (NS) is one of the most studied and with the highest social impact. In the last few years, many new aspects of the pathogenesis of the disease have been unveiled. Now it is clear that, at least in the genetic and familiar forms, but also in many acquired cases, the filtration defect of NS depends on the quantitative and/or qualitative alteration of the podocyte slit-diaphragm protein components. The slit-diaphragm is a very specialized cell junction, with a selective filtration capacity, which preserves the organism from losing important proteins into the preurine filtrate. The major culprits have been identified in nephrin (NPHS1) and podocin (NPHS2) [8–13], among the many others. Nephrin is a large membrane protein with an IgG like ectodomain, belonging to the IgG superfamily proteins, while podocin is an integral membrane protein with one transmembrane domain and a C-terminal cytoplasmic tail. The nephrin transmembrane domain and most of the cytoplasmic tail are homologous to the corresponding regions of the stomatin family proteins [14]. Nephrin and podocin interact physically and functionally, linking the cell membrane to the cytoskeleton and directing its reorganization [15, 16]. Most of the genetic forms of NS seem to carry mutations impairing the expression or the function of these two proteins, although impairment of all the other slit-diaphragm components can induce several degrees of proteinuria, up to severe NS or focal segmental glomerulosclerosis (FSGS) [17–19]. As a result, the glomeruli become unable to perform their selective filtration function, leading to massive proteinuria. On the other hand, also external causes can induce damage to the podocytes, like infective, metabolic, and immunological agents. In these cases, as well, the slit-diaphragm seems to be the pathogenetic target. Besides the loss of the filtration function, NS develops a progressive and persistent fibrosis leading to the loss of functional glomeruli. So far, fibrosis has been mainly seen as consequence of the inflammatory and immunological response due to the involvement of nonresident cells, often present in the disease. Still, many investigators agree that these factors can account for no more than 50–60% of the observed fibrosis. In the case of tubular interstitial sclerosis, the other major outcome of many renal pathologies, the fibrosis has been partially attributed to the intrinsic ability of the renal tubular epithelial cells to undergo EMT [20–22]. Usually, this is seen as consequence of increased activity of TGF-*β*1 [23, 24] and other cytokines produced during these diseases. Many theories and *in vivo* models have been proposed in this regard. Among all, a very reasonable and suggestive hypothesis is that the podocyte itself might be active producer of the sclerotic tissue in the NS, by undergoing EMT (personal observation) [25]. Other earlier and more recent investigations, respectively, showed the ability of the podocyte to express low levels of *α*-SMA and undergo EMT [26–28] although, in the some cases, the authors referred to it as glomerular epithelial cells, not univocally identifying them as podocytes [29]. Thus, we reasoned that, if the slit-diaphragm were pathologically altered, like for the nephrin genetic deficiency, this would lead to an impairment of the cell contact machinery. The loss of the cell contacts is, indeed, one of the triggering mechanisms of EMT [30–32]. Therefore, the nephrin deprived podocyte itself might undergo EMT, contributing to the consequent sclerotic outcome. In the present paper, we tested our hypothesis and showed that a conditionally immortalized human podocyte cell line is able to undergo EMT upon TGF-*β*1 treatment, like previously shown in a murine model [28].

## 2. Materials and Methods

### 2.1. Cell Lines

Conditionally immortalized wild type and nephrin-mutated human podocytes cell lines (kindly supplied by Saleem, MA, Academic and Children's Renal Unit, University of Bristol, Bristol, UK) were developed by transfection with the temperature-sensitive SV40-T gene, as previously described [33, 34]. The nephrin-mutated podocyte cell line was deprived of nephrin by genetic manipulation that inserted a constitutive 121delCT frame shift mutation in nephrin transcript [35]. Both cell lines proliferate at the “permissive” temperature (33°C). After transfer to the “nonpermissive” temperature (37°C), they enter growth arrest and express markers of differentiated *in vivo* podocytes. The cells were grown at the appropriate temperature, with 5% CO_2_ in a humidified incubator in a growth medium composed of RPMI 1640, 10% fetal bovine serum (FBS), 1% glutamine, 1% antibiotics, 10 mg/mL insulin, 5.5 mg/mL transferrin, and 5 ng/mL sodium selenite (purchased as a mix from Sigma-Aldrich, St. Louis, MO, USA, product code I-3146). Where indicated, the cells were starved for 48 hr in 1% FBS and treated with 5 ng/mL of TGF-*β*1 (cod. 100-21), which was purchased from Pepro Tech, Rocky Hill, NJ, USA. In the experiment in absence of calcium, the cells were grown as indicated above but in calcium-free RPMI 1640 (Gibco, OH, USA) as medium and 1% FBS.

### 2.2. Protein Extraction and Western Blots

Total cell lysates were prepared in RIPA buffer (50 mM Tris-HCL, 150 mM NaCl, 1% Triton X-100, and 1 mM ethylen-diamine-tetra-acetic acid, EDTA), added with a protease cocktail and the phosphatase inhibitor cocktails I and II (Sigma, MO, USA). The lysates were cleared by 30 min centrifugation at 20,000 ×g. Typically, 30 *μ*g of the total cell lysates was separated on SDS-PAGE. Where indicated, the cell extract was fractionated in membranes, cytoplasm, and nuclei as following. After extensive washing in PBS, the cells were scraped in isotonic buffer (40 mM HEPES, pH 7.5, 5 mM MgCl_2_, and protease and phosphatase inhibitors). Then they were passed several times through a 21-gauge needle and frozen and thawed for three times. The nuclear fraction was pelleted at 720 ×g for 10 min at 4°C and resuspended in RIPA buffer. The supernatant was centrifuged at 30,000 ×g × 30 min at 4°C, and the pellet containing the membrane fraction was resuspended in RIPA buffer. The supernatant contained the cytoplasm fraction. The protein concentration was determined using a Blue-Coomassie based assay [36]. Then, the proteins were blotted to a PVDF Immobilon-P membrane, which was previously prepared according to the manufacturer's protocol (Millipore, Bedford, MA, USA). After blocking in TBS/1% BSA (0.1 M NaCl, 0.05 M Tris-HCl, pH 7.4, and 1% BSA, pH 7.5) for 2 hr, the membrane was incubated overnight or for at least 3 hr with the indicated antibodies. The antibody providers are indicated in the relative figures. Then, the blots were incubated with the proper alkaline phosphatase conjugated secondary antibodies and developed with nitro blue tetrazolium chloride/5-bromo-4-chloro-3-indolyl phosphate (NBT/BCIP) reagents (Roche Diagnostics GmbH, Mannheim, Germany).

### 2.3. Cell Immunofluorescence (Figure 2)

The cells were grown to subconfluence on cover glass (BDH, Milan, Italy) fixed with 4% paraformaldheyde. The cells were permeabilized with 0.1% saponin. The blocking was obtained by incubating the slides in 1% BSA. The primary antibodies were used at a 1 : 10 to 1 : 20 dilution in the blocking solution as indicated in the relative figures. The goat Snai-1 (homologous of Drosophila Snail) (sc-10433) and anti-Fsp1 (also known as Mts-1) (sc-19949) antibodies were purchased from Santa Cruz Biotechnologies, (Santa Cruz, CA, USA). The secondary antibodies were conjugated with Alexa Fluor 488 dye (Molecular Probes, Eugene, OR, USA) and used at 1 : 100 dilution. The confocal laser-scanning microscopy was performed using a Focal Scanning System Laser Co MRC 1024 ER (BioRad, USA). The nuclei were previously stained with propidium iodide.

### 2.4. Tissue Immunofluorescence (Figure 9)

Expression levels of *α*-SMA, WT-1, nephrin, and proteins were evaluated by indirect immunofluorescence and confocal microscopy analysis on renal biopsy from patient with nephrotic syndrome of Finnish type homozygous for p.S569R (c.1707C>A) NPHS1 mutation and from wild type (WT) subject. Briefly, the slides were incubated overnight with the primary antibodies at 4°C (mouse anti-*α*-SMA, Sigma-Aldrich, 1 : 100; rabbit anti-WT-1, sc-192, Santa Cruz Biotechnologies, 1 : 100; guinea pig anti-nephrin, Progen Biotechnik, 1 : 100), washed in PBS, and then incubated with goat anti-mouse IgG-Alexa Fluor 488, goat anti-rabbit IgG-Alexa Fluor 555, and chicken anti-guinea pig IgG-Alexa Fluor 488, respectively. The slides, mounted with an antifading aqueous medium (Gel/Mount, Biomeda), were examined under a fluorescence microscope equipped with appropriate filters (Leica TCS SP5; Leica, Wetzlar, Germany). Confocal images were taken at 500 nm intervals through the *z*-axis of the section, encompassing a total of 4 *μ*m in depth. Images from individual optical planes and multiple serial optical sections were analyzed, and the images were sequentially scanned. Negative controls were performed by replacing the primary antibody with nonimmune serum at equivalent concentration. Each experiment was carried out three times.

### 2.5. Nuclear Extracts

The cells were collected by scraping with a rubber policeman, after previous wash with PBS. The cells were pelleted at 600 g for 10 min, resuspended in 200 *μ*L/100 mm plate of 20 mM HEPES, pH 7.9, 1 mM EDTA, 1 mM dithiothreitol, and protease and phosphatase mix (see Section 2.2), and kept at 4°C for 15 min. The cell suspension was then added with 1/4 vol. of 1% NP40 to obtain a final concentration of 0.2% NP40 and incubated at 4°C for 15 min. The cell lysate was then centrifuged at 600 g for 15 min. The pellet (P1) mostly containing the unbroken nuclei was resuspended in 1 vol. of BLS (20 mM HEPES, pH 7.9, 1.5 mM MgCl_2_, 0.2 mM EDTA, 0.5 mM dithiothreitol, 0.5 mM PMSF, 80 mM NaCl, and 25% glycerol). After extensive mixing, 1 vol. of BHS (as in BLS, but containing instead 0.9 M NaCl) was slowly added and incubated at 4°C for 30 min under moderate shaking. P1 was centrifuged at 16,000 g for 30 min. The protein concentration was determined using a Blue-Coomassie based assay [36].

### 2.6. Electrophoresis Mobility Shift Assay (EMSA)

The assay was performed as previously described [37] using 2–5 *μ*g of nuclear extract proteins in a total volume of 20 *μ*L containing 20 mM Tris-HCl, pH 7.5, 0.1 M NaCl, 0.35 mM dithiothreitol, 0.5 mM EDTA, 0.5 mM PMSF, 10% glycerol, and 0.5 *μ*L of 2 mg/mL of double stranded poly(dI-dC) (1 *μ*g/reaction). The sequences of the oligonucleotides used as double-strand probe was the following: NF-*κ*B plus: 5′-AGT TGA GGG GAC TTT CC CAG GC-3′; NF-*κ*B plus: 5′- gCC TGG GAA AGT CCC CTC AAC T-3′. The oligonucleotides utilized were labeled at their 5′ ends with *γ*−(^32^P)ATP (NEN Life Science) using T4 polynucleotide kinase. The rabbit anti-p65 (C-20) antibody was purchased from Santa Cruz Biotechnologies. The binding to the double-stranded oligonucleotides was performed by incubating at 4°C for 30 min 10 ftmoles of ^32^P-labeled probes with the previous nuclear extract. Where indicated, 1 *μ*L of antibody anti-p65 was preincubated at 4°C for 30 min with the nuclear extract. The DNA-protein complexes were separated by electrophoresis on a 5% polyacrylamide gel and detected by autoradiography of the dried gel.

## 3. Results

**Figure 1:**
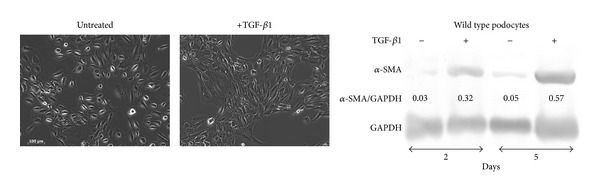
Phenotypic features of wild type (WT) human podocyte cell lines and *α*-SMA expression: effects of TGF-*β*1 treatment. (a) The cells were grown as indicated in Section 2. Prior to the TGF-*β*1 incubation, the cells were starved for 48 hr. The pictures show the cells after 48 hr of TGF-*β*1 treatment used at 5 ng/mL. The optical images were obtained with a Will (Wetzelar) inverted microscopy at a 10x enlargement. (b) Western blot detection of *α*-SMA. Typically, 30 *μ*g of total cell lysates was loaded on a 10% polyacrylamide gel. Western blot is described in Section 2. GAPDH expression was used to normalize protein loading. The anti-*α*-SMA mouse monoclonal antibody was purchased from Sigma-Aldrich (St. Louis, MO, USA). The anti-GAPDH (sc-25778) rabbit polyclonal antibody was purchased from Santa Cruz Biotechnologies, (Santa Cruz, CA, USA). To quantify the protein bands, we scanned the blots and performed a densitometry analysis, using the software NIH ImageJ v. 6.4 (freeware, NIH, MD, USA). The figure shows a typical experiment of at least three performed under the same conditions.

**Figure 2:**
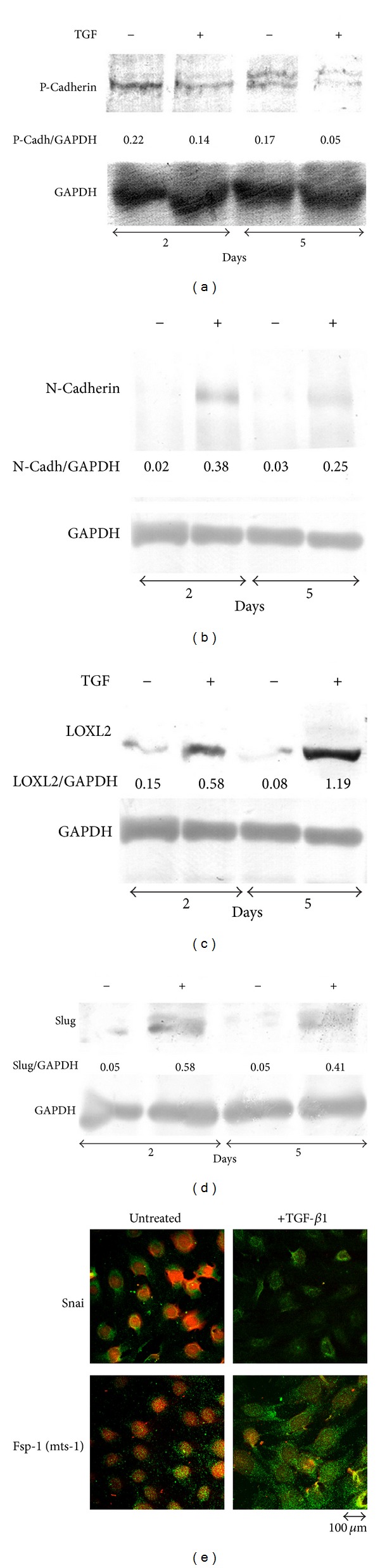
Analysis of additional EMT marker expressions in wild type human podocytes, upon TGF-*β*1 treatment. The cells were treated as indicated in Figure 1. (a) The rabbit anti-P-cadherin (sc-7893), (b) N-cadherin (sc-7939), (d) anti-Slug (sc-15321) and polyclonal antibodies were purchased from Santa Cruz Biotechnologies (Santa Cruz, CA, USA). (c) The rabbit anti-LOXL2 (A01) was purchased from Abnova (Taiwan). To quantify the protein bands, we scanned the blots and performed a densitometry analysis, using the software NIH ImageJ v. 6.4 (freeware, NIH, MD, USA). (e) Confocal laser scanned immunofluorescence of Snail-1 and Fsp-1 expressions. The cells were treated as indicated and prepared for immunofluorescence analysis as described in Section 2. The goat anti-Snail-1 (sc-10433) and anti-Fsp1 (also known as Mts-1) (sc-19949) antibodies were purchased from Santa Cruz Biotechnologies, (Santa Cruz, CA, USA). The secondary antibodies were conjugated with Alexa Fluor 488 dye (Molecular Probes, Eugene, OR, USA). Nuclei were previously stained with propidium iodide. The figure shows a typical experiment of at least three performed under the same conditions.

**Figure 3:**
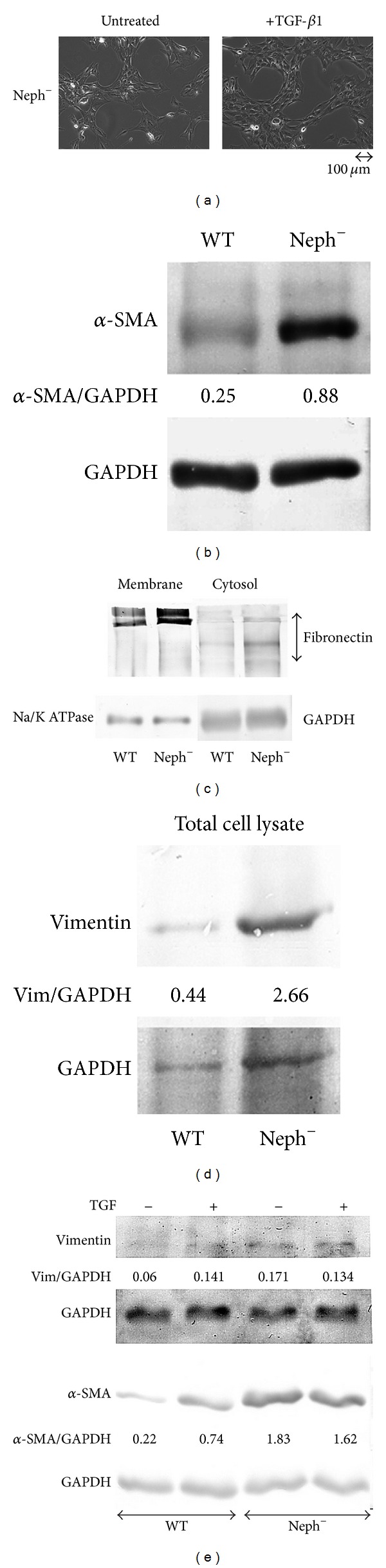
Phenotypic features of nephrin-mutated podocytes and EMT marker expression in human wild type and nephrin-mutated podocytes: effects of TGF-*β*1 treatment. (a) The cells were grown as indicated in Section 2. Prior to the TGF-*β*1 incubation (a) and (e), the cells were starved for 48 hr. The pictures show the cells after 48 hr of TGF-*β*1 treatment used at 5 ng/mL. The optical images were obtained with a Will (Wetzelar) inverted microscopy at a 10x enlargement. (b) *α*-SMA detection in wild type (WT) and nephrin deprived human podocytes (Neph^−^). The anti-*α*-SMA mouse monoclonal antibody was purchased from Sigma-Aldrich (St. Louis, MO, USA). (c) Detection of fibronectin bound to the membrane fraction and in the cytoplasm. Na-K-ATPase-*α*1 was used as cell membrane marker to normalize the protein loading. Mouse monoclonal anti-fibronectin (sc-18825), goat anti-Na-K-ATPase-*α*1 (sc-16041) polyclonal antibodies were purchased from Santa Cruz Biotechnologies (Santa Cruz, CA, USA). (d) Detection of vimentin in the total cell lysate. The goat anti-vimentin (sc-7557) was purchased from Santa Cruz Biotechnologies (Santa Cruz, CA, USA). (e) Effects of TGF-*β*1 on vimentin and *α*-SMA expression in the indicated podocyte cells. To quantify the protein bands, we scanned the blots and performed a densitometry analysis, using the software NIH ImageJ v. 6.4 (freeware, NIH, MD, USA). The figure shows a typical experiment of at least three performed under the same conditions.

**Figure 4:**
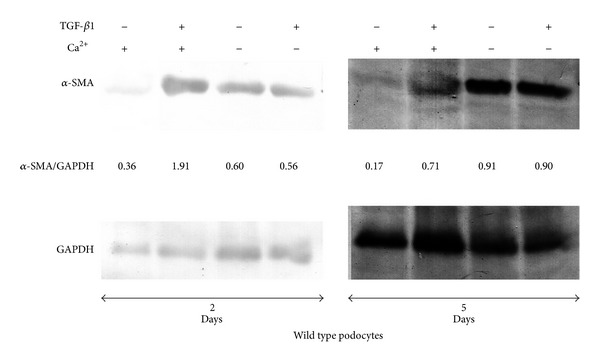
Effects of Ca^2+^ deprivation and TGF-*β*1 on *α*-SMA expression in wild type podocytes. The cells were exposed to the indicated treatment and processed for western blot as specified in Section 2. The antibodies used are described in the previous figures. To quantify the protein bands, we scanned the blots and performed a densitometry analysis, using the software NIH ImageJ v. 6.4 (freeware, NIH, MD, USA). The figure shows a typical experiment of at least three performed under the same conditions.

**Figure 5:**
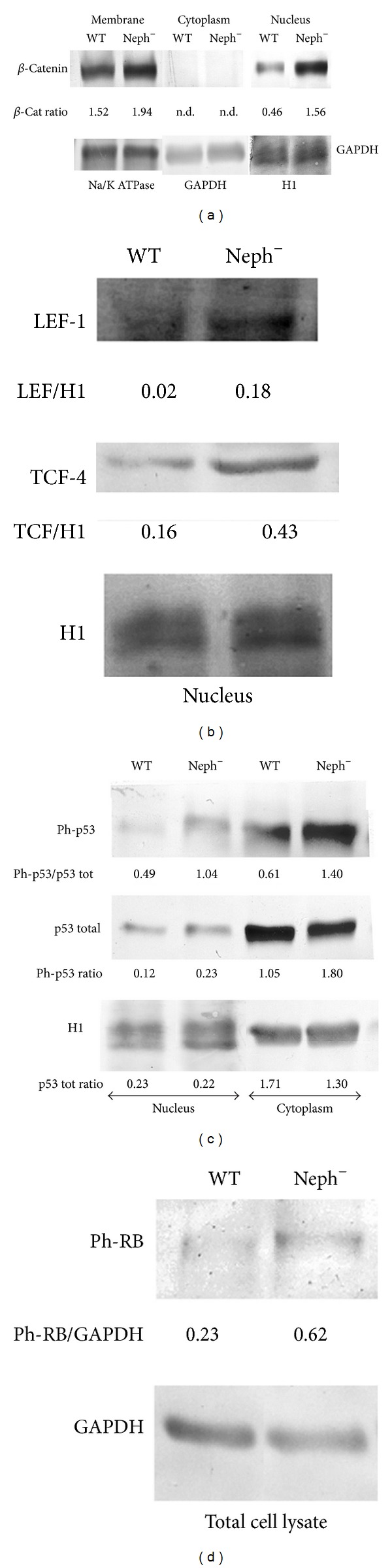
*β*-Catenin, TCF-4, LEF-1, p53, and pRB distribution and expression in wild type and nephrin-mutated podocytes. The cells were lysated, fractionated and processed for western blot as described in Section 2. (a) Distribution and expression of *β*-catenin in cell membranes and nuclear compartment. The goat anti-*β*-catenin (sc-1496) antibody was purchased from Santa Cruz Biotechnologies (Santa Cruz, CA, USA). (b) Expression of LEF-1 and TCF-4 in nuclear compartment. The goat anti-LEF-1 (sc-8591) and anti-TCF-4 (sc-8631) antibodies were purchased from Santa Cruz Biotechnologies (Santa Cruz, CA, USA). (c) Phosphorylated and total p53 distribution and expression in nucleus and cytoplasm. The mouse monoclonal anti-p53 IC12 (no. 2524) and the rabbit polyclonal anti-Phospho-p53 (ser15) (no. 9284) antibodies were purchased from Cell Signaling Technologies (Boston, MA, USA). (d) Phosphorylated-RB expression in total cell lysate. The rabbit anti-phospho-RB (ser780) (no. 9307) antibody was purchased from Cell Signaling Technologies (Boston, MA, USA). To quantify the protein bands, we scanned the blots and performed a densitometry analysis, using the software NIH ImageJ v. 6.4 (freeware, NIH, MD, USA). Where the relative amount of protein is indicated as generic “ratio” instead of ratio to a specific housekeeping protein, it is because the protein quantization referred to the different cell compartment housekeepers indicated at the bottom of each blot. The figure shows a typical experiment of at least three performed under the same conditions.

**Figure 6:**
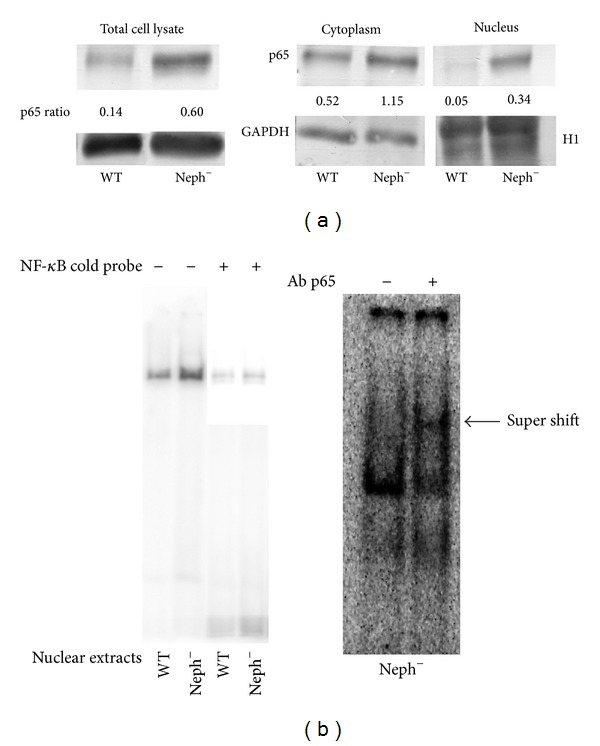
NF-*κ*B p65 expression, distribution, and EMSA for NF-*κ*B consensus in wild type and nephrin-mutated podocytes: (a) WT and Neph^−^ podocytes were grown, fractionated, and processed for western blot as described in Section 2. The rabbit anti-p65 (sc-372) polyclonal antibody was purchased from Santa Cruz Biotechnologies (Santa Cruz, CA, USA). The sheep anti-histone H1 (H5110) polyclonal antibody was purchased from US Biological (Swampscott, MA, USA). To quantify the protein bands, we scanned the blots and performed a densitometry analysis, using the software NIH ImageJ v. 6.4 (freeware, NIH, MD, USA). (b) Nuclear extracts from WT and Neph^−^ podocytes were prepared as indicated in Section 2. EMSA was performed using NF-*κ*B consensus [^32^P]-labeled double-strand oligonucleotide. The sequence of the probe and the EMSA technique are described in Section 2. The anti-p65 antibody is the same as in part A of the present figure, but 10 times more concentrated (sc-372X) (Santa Cruz Biotechnology, Santa Cruz, CA, USA). The figure shows a typical experiment of at least three performed under the same conditions.

**Figure 7:**
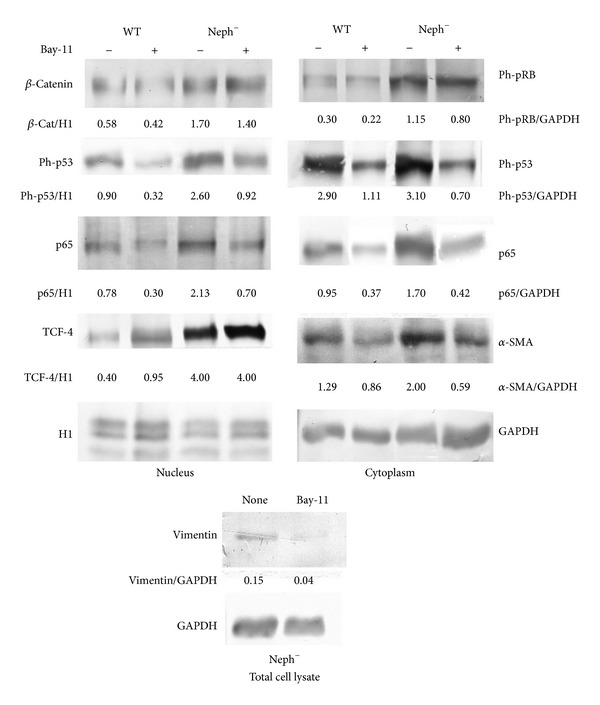
Effects of Bay-117082 on the distribution and expression of p53 and its phosphorylated form, phosphorylated pRB, *β*-catenin, a-SMA, vimentin, and p65 in wild type and nephrin-mutated podocytes. Where indicated, the cells were treated with Bay-117082 (Calbiochem, USA) at 0.5 mM for 4 days (in the figure indicated as Bay-11). Afterwards, they were lysated, fractionated, and processed for western blot as described in Section 2. The antibodies used have been described in the previous figures. To quantify the protein bands, we scanned the blots and performed a densitometry analysis, using the software NIH ImageJ v. 6.4 (freeware, NIH, MD, USA). The figure shows a typical experiment of at least three performed under the same conditions.

**Figure 8:**
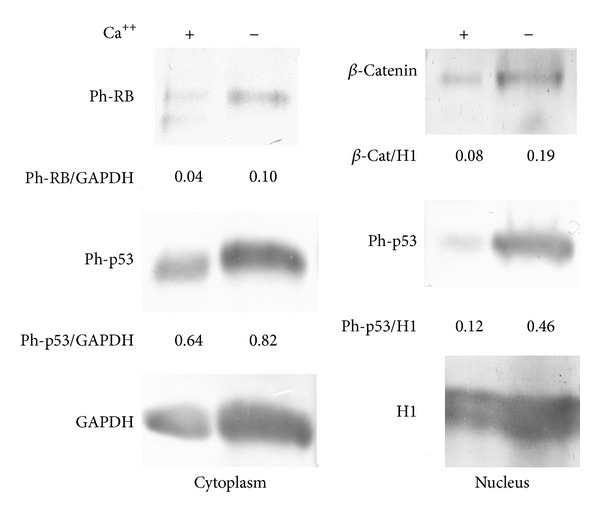
Growth of wild type podocytes in Ca^2+^-free medium induced the same effects of nephrin ablation on *β*-catenin, p53, and phospho-pRB expressions and distribution. The cells were lysated, fractionated, and processed for western blot as described in Section 2. The antibodies used have been described in the previous figures. To quantify the protein bands, we scanned the blots and performed a densitometry analysis, using the software NIH ImageJ v. 6.4 (freeware, NIH, MD, USA). The figure shows a typical experiment of at least three performed under the same conditions.

**Figure 9:**
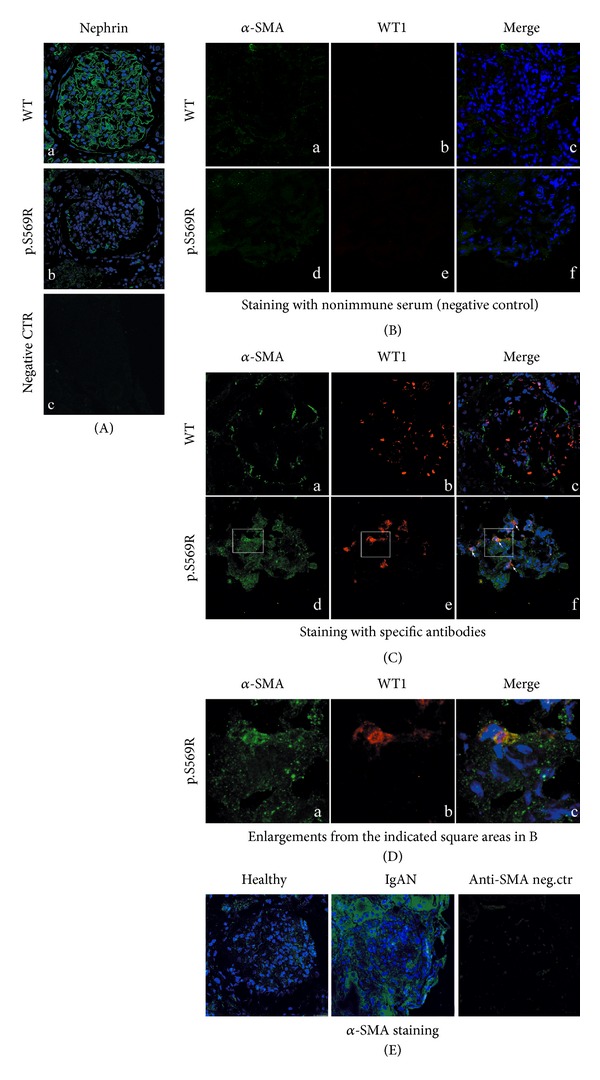
Nephrin and *α*-SMA expression in kidney biopsies from a normal individual and a patient carrying a Finnish mutation p.S569R (c.1707C>A) (NPHS1) and in IgAN. Immunofluorescence and confocal analysis were carried out as described in Section 2. (A) Nephrin expression in normal (WT) (a) and NPHS1 kidney (b). (c) shows the antibody negative control. (B) Negative control for *α*-SMA (a) expression and WT1 (b) antibodies and their merged signals (d). (C) *α*-SMA and WT1 expression in normal (WT) and NPHS1 kidney (WT a-b and p.S569R d-e) and their merged signal (c-f). *α*-SMA colocalizes with WT1 indicating its podocyte origin. (D) Enlarged particular of panel (c) showing the colocalization of *α*-SMA and WT1 in NPHS1 kidney tissue and their merged signals (a, b, and c). (E) *α*-SMA expression in healthy or IgA nephropaty (IgAN) renal biopsy. The technical details are described in Section 2. The figure shows a typical experiment of at least three performed under the same conditions.

In the present investigation, we used two conditionally immortalized human podocyte cell lines. One reproduced the normal podocyte features (wild type podocytes: WT) [33], while the other was genetically deprived of nephrin with a constitutive 121delCT frame shift mutation in nephrin transcript (nephrin-mutated podocytes: Neph^−^) [35]. The Neph^−^ clone showed in this paper is representative of other tested nephrin deprived clones, since they all gave similar results and the experiments were always performed with at least 3 other similarly obtained clones, to rule out a “casual clone effect”. Both lines were immortalized with a retroviral construct carrying the SV40 large *T* antigen gene containing both the tsA58 and the U19 mutations. The immature podocytes were able to differentiate into nonreplicating mature podocytes only at nonpermissive temperature. In Figure 1 (left panel), the effects of TGF-*β*1 treatment on wild type human podocytes are shown. Differentiation into a fibroblast-like phenotype is evident. Assuming an EMT process behind such a phenotype, we tested the podocytes for the expression of *α*-SMA. The western blot in the right panel of Figure 1 shows that TGF-*β*1, indeed, induced a strong 10-time increase of *α*-SMA already after 48 hr of treatment and was persistent even after five days. Noteworthy, it is the presence of discrete amounts of *α*-SMA in the untreated podocytes, as previously described in these cells [38]. Another report showed that *α*-SMA is expressed in primary murine podocytes and seems to play a role in their maturation [39]. Yet, although constitutively expressed, in our experiments, TGF-*β*1 significantly triggered *α*-SMA expression in an EMT process fashion. To better define whether we were in the presence of a typical EMT, we searched for a set of other specific EMT markers. Therefore, we looked at the downregulation of P-cadherin, typically expressed in podocytes, instead of the classical E-cadherin. In Figure 2(a), we showed that P-cadherin was almost completely absent after TGF-*β*1 treatment, being strongly downregulated already after 48 hr. In regard to cadherin behavior, an interesting finding was the switch to N-cadherin (Figure 2(b)), which is often observed during EMT occurring in metastasizing epithelial tumors [40]. Screening for other EMT markers, we looked at LOXL2, a member of LOX family [41, 42], recently identified as an upstream inhibitor of the proteasome-dependent degradation of Snail [43, 44], which in turn, down regulates E-cadherin. In this case too, we found an important upregulation (Figure 2(c)). The same increased expression was detected for Slug, transcriptional partner of Snail-1 [45, 46] (Figure 2(d), right panel).  Figure 2(e) also showed confocal images of Snail-1 and Fsp-1 (mts-1), specific fibroblast marker. The figure showed their expressions in basal conditions or after 5 days of TGF-*β*1 treatment and confirmed that they were upregulated as well. So all the main actors of the EMT initiation were affected. Taken together, these results suggest that we were in presence of a typical epithelial-mesenchymal transition. Since the typical nephrotic syndrome is characterized by a qualitative and/or quantitative impairment of proteins of the slit-diaphragm, mainly nephrin and/or podocin, we thought that the syndrome might configure a condition of cell contact loss, which by itself has been described as a possible EMT trigger [30, 31]. To test our hypothesis, we analyzed the nephrin-mutated podocytes for the same myofibroblast markers, with and without TGF-*β*1 treatment. First, we noticed that they significantly differed at a phenotypic level from the wild type podocytes (compare Figure 1, right panel WT and Figure 3(a) Neph). They showed a more elongated fibroblast-like shape. Western blots indicated a much higher expression of *α*-SMA as compared to the wild type podocytes (Figure 3(b)). In addition, fibronectin and vimentin were constitutively highly expressed (Figures 3(c)-3(d)), while they were almost absent or weakly expressed in the control cells. Interestingly, in the same figure (panel (e)) we show that these typical mesenchymal markers, like vimentin and *α*-SMA, failed to be upregulated by TGF-*β*1, probably because they are already expressed at high levels, compared to the ones achieved by the wild type podocytes when triggered by this cytokine. Taken together, this evidence suggests that the nephrin deficiency itself induced an EMT process. To test whether those features depended on the rupture of the homophilic contacts between podocytes, due to the absence of nephrin, we reproduced a similar condition in the wild type podocytes, by growing them in Ca^2+^-free medium. According to our hypothesis, the wild type podocytes in absence of Ca^2+^ showed a significant upregulation of *α*-SMA (Figure 4). It was already evident after 2 days of Ca^++^ deprivation, reaching the same extent of TGF-*β*1, or even higher, after 5 days (from about 2 times to 5 times after 2 and 5 days). The TGF-*β*1 did not affect the already high level of *α*-SMA. Then, we decided to study the consequences of nephrin deficiency on the podocyte intracellular signaling. Since the EMT induces loosing of cell contacts, of which the downregulation of E-cadherin is the most typical consequence (P-cadherin in the podocytes case), we looked at the signaling downstream of it. We focused first on *β*-catenin, a direct cadherin target. As evinced by its expression and cellular distribution, we found, indeed, a significant activation of *β*-catenin.  Figure 5(a) showed, in fact, a nuclear increase of *β*-catenin translocation and its overall higher expression (Figure 5(a): Membrane + Nucleus). However, the activation of *β*-catenin was not induced by TGF-*β*1 in wild type podocytes (data not shown), in contrast with previous reports [31]. Based on these findings, we analyzed the nuclear levels of TCF-4 and LEF-1, two of the main transcription factors cooperating with *β*-catenin at transcription level. In Figure 5(b), it is shown that nuclear TCF-4 and LEF-1 were increased, respectively, three and nine times. Wondering about the consequences at cell cycle level of E-cadherin-*β*-catenin signaling perturbation, we investigated the functional status of two important tumor suppressor and cell proliferation regulators, often acting in coordination: p53 and pRB.  Figure 5(c) shows that p53 phosphorylated form is increased, both as fraction of total p53 (phospho-p53/p53 total) and as absolute amount, both in the cytoplasm and nucleus (phospho-p53/GAPDH or /Histone H1). However, the expression of total p53 (phosphorylated and unphosphorylated) between the wild type and nephrin-mutated podocytes was not significantly changed (p53 total/GAPDH or /histone H1). Panel (d) of the same figure shows also a sensible increased phosphorylation of pRB in nephrin-deficient podocytes, meaning an overall less active status. Investigating other main signaling pathways, in agreement with a recent report [47], we found also a significant upregulated expression and activation of NF-*κ*B p65 (Figure 6(a), left panel). In fact, most of the increase of p65 is localized in the nuclear compartment (Figure 6(a), right panel). In the same figure, the EMSA for the NF-*κ*B consensus sequence is also shown, confirming an increase of p65 binding to its DNA target in nephrin-mutated versus wild type podocytes (Figure 6(b), left panel). In Figure 6(b), right panel, the p65 identity in the major DNA-protein complex detected is also confirmed, since a specific anti-p65 antibody induced a supershift of the radiolabelled band. Since NF-*κ*B is central to many transduction pathways, we wondered whether the results regarding p53 and pRB, and maybe *β*-catenin, were in some way dependent on NF-*κ*B activation. To this purpose, we blocked the I*κ*B-*α* proteasome-dependent degradation with Bay-117082, an inhibitor of I*κ*B-*α* phosphorylation [48], inducing, therefore, degradation of NF-*κ*B. In these conditions, we screened both podocyte cell lines for *β*-catenin, Ph-p53, Ph-RB, and some of the mesenchymal markers which are overexpressed in nephrin-mutated podocytes. In Figure 7, it is shown that Bay-117082 lowered p65 expression as expected, but also p53 phosphorylation was diminished and *α*-SMA was down-regulated in both podocyte cell lines. In the figure, we showed that even vimentin level was almost shut down by the treatment in the nephrin-mutated podocytes, indicating a sort of reversion of EMT. Conversely, pRB phosphorylation and *β*-catenin nuclear translocation remained unaffected, as well as the nuclear level of TCF-4, a *β*-catenin target. Strangely, Bay-117082 upregulated TCF4 in the wild type podocytes. We do not have an explanation for that at this moment. Thus, not all the altered intracellular signaling of the nephrin-mutated podocyte can be ascribed to NF-kB activation, since *β*-catenin and pRB are not affected. It does, though, have an effect on the mesenchymal transformation, since *α*-SMA and vimentin are down-regulated upon its inhibition. This has been described in other models [49–52]. To reinforce our original hypothesis, we tested whether also these altered pathways could be reproduced in the wild type human podocytes by Ca^2+^ deprivation.  Figure 8, indeed, shows that *β*-catenin nuclear translocation rate and p53 and pRB phosphorylation levels mirrored their behavior in nephrin-mutated podocytes. At this point, it is evident that nephrin deprivation and, therefore, cell contact rupture must induce a wide and complex series of cross-interacting signals probably EMT-dependent. A natural question arising from this study is whether these *in vitro* findings reflected a physiopathological occurrence in human chronic nephropathies, dependent on nephrin impairment, like for NPHS1. To answer the question, we analyzed by immunofluorescence and confocal microscopy renal biopsies from patients with nephrotic syndrome of Finnish type. The one shown here is homozygous for p.S569R (c.1707C>A) NPHS1 mutation. The results in Figure 9 demonstrated that *α*-SMA was highly expressed in the glomeruli of the tested NPHS1 biopsy, as compared to a kidney biopsy from a normal tissue. In Figure 9, panel (A) (compare (a) versus (b)), we showed the absence of nephrin expression in the NPHS1 glomeruli, as expected. The panel (C) of the same figure showed the colocalization of *α*-SMA with WT1, typical podocyte marker, to indicate its specific podocyte expression (compare (a), (b), and (c) versus (d), (e), and (f)). Such a colocalization is better shown in panel (D), which is an enlargement of a particular of panel (C). In panel (B) of Figure 9 the negative control reaction for the immunofluorescence of *α*-SMA and WT1 is shown, as indicated. In panel (E) of the same figure, we showed the expression of *α*-SMA in a different renal pathology that is not based on nephrin impairment, the IgA nephropathy. In this case, the expression was much less pronounced as compared to the biopsy of a healthy subject and, more importantly, did not seem to affect the podocyte area of the glomerulus. Therefore, the bioptic analysis gave further support to our cellular studies.

## 4. Discussion

Overall, our investigation demonstrated that human podocytes lines are prone to undergo EMT. They responded to the most classical EMT inducer, the TGF-*β*1. The P-cadherin was down-regulated, and the expression of *α*-SMA, typical myofibroblast marker, was upregulated and so all the major players of EMT. Our study agrees with other investigator findings in similar mouse cell systems [28, 29, 39]. We believe that our findings corroborate the hypothesis of podocyte EMT as a putative mechanism underlying the sclerotic reactions that characterize human nephrotic syndromes and other glomerular-based pathologies. More interestingly, we showed that genetic ablation of nephrin induced a constitutional EMT as well, with most of the above indicated molecular hallmarks. Interestingly, TGF-*β*1 barely affected, if at all, the expression of mesenchymal markers in nephrin-mutated podocytes, just as expected in a myofibroblast phenotype. These results prompted us to test the hypothesis that the impairment of the slit-diaphragm, and in general, of the cell contacts, might have a role in nephrin deficiency-dependent EMT. In fact, the rupture of the intercellular junctions by Ca^2+^ deprivation induced, in the human wild type podocytes, phenotypic and molecular expression changes very similar to the EMT observed in the nephrin-mutated podocytes. This finding might explain as well glomerulosclerosis occurring in nephrotic syndromes in which components of the slit-diaphragm other than nephrin are absent or functionally impaired. A more detailed investigation on the molecular consequences of nephrin ablation showed complex alterations of critical pathways, involving *β*-catenin, NF*κ*B, p53, and pRB. Considering the indubitable upsetting of the slit-diaphragm induced by nephrin ablation and EMT-dependent downregulation of P-cadherin, we postulated that *β*-catenin, among others, was likely the most affected protein in the network between the cell membrane and the cytoskeleton. In fact, many investigations reported concomitant *β*-catenin activation upon E-cadherin knocking down or inhibition [53–55]. Although primary *β*-catenin activation seems to be able to indirectly downregulate E-cadherin, by activating Snail, while it is known that Snail, in turn, can also downregulate nephrin [56], in our model, it is more likely that *β*-catenin activation is secondary to the cell contact impairment and consequent loss of cadherin. However, Wnt-1/*β*-catenin pathway has been recently highly considered in the podocytopathies as a new important pathogenetic or favoring factor to be taken into account [57, 58]. Definitely, it is more difficult to understand the p53 activation and pRB downmodulation by hyperphosphorylation. We do not know yet what their role is in the economy of this kind of podocyte damage, mainly because we did not find any apoptosis increase. However, the absence of apoptosis could still be explained by the survival signaling coming from *β*-catenin activation pathway. As matter of fact, a case of p53 activation and increase of pRB phosphorylation has been described as dependent on nutlin-3, a p53 indirect activator [59]. Moreover, previous observations indicated *α*-SMA to be positively regulated by p53 [60]. Also, p53 activation in aggressive fibromatosis together with *α*-SMA expression [61], and in another case, RB depletion, both induced E-cadherin downregulation and EMT [62]. Adding further complexity to the observed pathway picture, we confirmed the activation of NF-*κ*B, in agreement with a recent investigation on the same nephrin-deficient cell line [47]. Based on the presented findings, we would like to express a different point of view at this regard, since we believe that NF-*κ*B activation is more likely dependent on the EMT/P-cadherin downregulation process, as previously noted in a model of malignant melanoma [51], rather than being induced directly by nephrin ablation. However, Hussain et Al. [47], while finding a NF-*κ*B activation, did not describe a nephrin deficiency-dependent EMT, like in our case. As matter of fact, NF-*κ*B must play a role in what we observed. In fact, Bay-117082-dependent inhibition of NF-*κ*B affected negatively p53 phosphorylation, as well as vimentin and *α*-SMA expressions. We are in presence of a complex signaling network that must be triggered at the slit-diaphragm level, but it is hard to say what is due just to nephrin absence and what is dependent on the cell contact impairment as whole. It could well be that nephrin ablation on one side and cell contacts loss on the other trigger a cascade of overlapping and interfering signals, probably orchestrated by the EMT process. Our *in vitro* study found also confirmation in kidney biopsy from a patient carrying a NPHS1 mutation. In fact, we found an overexpression of *α*-SMA at podocytes level. This is very strong evidence that the *in vivo* pathology follows a pathway that is not different from what we observed *in vitro*. Nevertheless, we are fully aware that more cases of NPHS1 should have been analyzed, but due to the difficulties to find those cases, we were not able to perform more immunofluorescence experiments. Though, it is quite suggestive that only the nephrotic syndrome caused by nephrin deficiency showed high levels of *α*-SMA, while in a IgA nephropathy biopsy [63], the slight increase of *α*-SMA was mainly detected outside the glomerular area. Finally, we showed that Ca^2+^ deprivation reproduced the described pathway alterations even in the wild type podocytes. Although we are aware that Ca^2+^ deprivation might induce more complex metabolic effects, the impairment of the cell contacts is one of the most evident consequences, and this method has been previously used to the same purpose [30, 31]. Therefore, we feel that this latter result is reinforcing our hypothesis that the cell-cell interactions might play an important and probably primary role in the phenomena here described.
